# Gonadotropin Stimulation Reduces the Implantation and Live Birth Rate but Not the Miscarriage Rate of Embryos Transferred When Compared to Unstimulated In Vitro Fertilization

**DOI:** 10.1007/s43032-022-01016-8

**Published:** 2022-06-29

**Authors:** Vera Ruth Mitter, Flavia Grädel, Alexandra Sabrina Kohl Schwartz, Michael von Wolff

**Affiliations:** 1 Division of Gynecological Endocrinology and Reproductive Medicine, Bern University Hospital, Inselspital Bern, University of Bern, Friedbühlstrasse 19, 3010 Bern, Switzerland; 2 Centre for Fertility and Health, Norwegian Institute of Public Health, Oslo, Norway; 3 Faculty of Medicine, University of Bern, Bern, Switzerland; 4 Division of Reproductive Medicine and Gynaecological Endocrinology, Cantonal Hospital Lucerne, Women’s Hospital, Lucerne, Switzerland

**Keywords:** Assisted reproductive technologies, Implantation, Natural cycle IVF, Gonadotropins, Clinical pregnancy, Live birth

## Abstract

Research suggests that gonadotropin stimulation in in vitro fertilization (IVF) treatment affects embryo quality and the endometrium that might influence embryo implantation, placentation and establishment of a viable pregnancy. We assessed the impact of gonadotropin stimulation on implantation, live birth and miscarriage rates per transferred embryo by comparing stimulated and unstimulated IVF treatment. In a cohort of 728 couples, 1310 IVF cycles with successful embryo transfer were analysed; 857 cycles were stimulated with gonadotropins > 75 IU/day (333 poor responder < 4 oocytes; 524 normal responders), and 453 were unstimulated. In total, 1913 fresh cleavage-stage embryos were transferred. Zygote but no embryo selection was performed, and supernumerous zygotes were vitrified. The implantation rate was defined as number of sonographically detected amniotic sacs; live birth rate as number of children born per transferred embryo. Modified mixed effect Poisson regression was used to account for the dependency of cycles and embryos within the same women and the same transfer cycle. Adjustments were made for maternal age, parity, primary or secondary infertility and indication for IVF. Per transferred embryo, implantation rates (rate ratio (RR) 1.37; 95% CI 1.04–1.81; *p* = 0.028; aRR 1.42; 95% CI 1.10–1.84; *p* = 0.008) and live birth rates (RR 1.33; 95% CI 0.95–1.86; *p* = 0.093; aRR 1.38; 95% CI 1.01–1.88; *p* = 0.044) were higher in NC-IVF compared to cIVF normal responders. Miscarriage did not differ (RR 0.99; 95% CI 0.59–1.65; *p* = 0.965; aRR 0.90; 95% CI 0.52–1.53 *p* = 0.698). Similar results were obtained in poor responders. The study suggests an impact of gonadotropin stimulation on the implantation potential of embryos.

## Introduction

In vitro fertilization (IVF) therapies were revolutionised by gonadotropins. Gonadotropins stimulate the growth of many follicles and allow the retrieval of several oocytes. In spontaneous menstrual cycles, only the largest follicle of a cohort survives and the development of the other follicles are inhibited by inhibin B, released from the largest follicle [1]. In gonadotropin-stimulated IVF therapies, this physiological regulatory effect is inactivated by the constantly high concentration of exogenous gonadotropins. The growing cohort of follicles leads to a polyfollicular ovarian response and the number of collected oocytes as well as the success of the IVF therapy per cycle is increased [2]. However, gonadotropin stimulation and the use of gonadotropin-releasing hormone (GnRH) analogues have shown to negatively affect cumulative livebirth rates in cycles where a large number of oocytes was collected at oocyte pick-up (OPU) [2, 3].

This might be due to some effects on follicular endocrinology. In gonadotropin-stimulated follicles, luteinising hormone (LH), androgen, oestradiol (E2) and anti-Mullerian hormone (AMH) concentrations are several-fold reduced at the time of follicle aspiration compared to naturally matured follicles [4]. This includes follicular AMH, which is known as a marker for the implantation potential of the oocyte in gonadotropin-stimulated IVF cycles [5, 6] as well as in unstimulated, natural IVF cycles (NC-IVF) [7]. Ovarian stimulation leads to alterations in the production of steroids and other hormones in luteal granulosa cells, which preserve the pregnancy [8].

Furthermore, gonadotropin seems to affect oocyte development. The energy of the oocyte is produced from pyruvate and cholesterol provided by the surrounding cumulus cells [9, 10]. The oocyte is in bidirectional communication with the cumulus cells to generate developmentally competent oocytes [11] and to resume meiosis induced by the LH surge.

These findings demonstrate that gonadotropin stimulation affects follicular physiology and possibly oocyte and embryo quality. A study investigating the intrinsic potential of oocytes to develop into a live-born child by using NC-IVF cycles found higher rates per oocyte retrieved when compared to gonadotropin-stimulated IVF, but the success was highly dependent on the woman’s age and decreased substantially above age 35 [12].

The aim of this study was to evaluate the impact of gonadotropin stimulation on the potential of fresh embryos to develop into a pregnancy and a live birth and its risk for a miscarriage. We compared embryos generated in conventionally gonadotropin stimulated IVF (cIVF) cycles with embryos generated in unstimulated NC-IVF cycles. Such a comparison is challenging, as in cIVF but not in NC-IVF embryo selection is performed which makes it impossible to compare the outcome of transferred embryos in both treatments. Therefore, in this study embryo selection was not performed taking advantage of the Swiss law that did not allow embryo selection before 2017. Fertilized oocytes had to be vitrified at the zygote stage.

## Materials and Methods

### Data Sources

Data on all cycles performed in Switzerland are collected by the Swiss ART registry “FIVNAT” for statistical purposes and for quality control [13]. Data on all cycles performed at the university hospital’s infertility centre between 2011 and 2016 (*n* = 3456) were extracted from “FIVNAT.” Thawing cycles (*n* = 910) and cycles without embryo transfer (*n* = 542) were excluded. Data on cycles with fresh embryo transfer leading to a pregnancy was provided by the Bern IVF Cohort (*n* = 311). In the Bern IVF Cohort, data on treatment and infertility reasons are documented in good quality and > 99% complete, based on medical history and delivery reports [14]. The cycles originating from both data sources were linked to identify duplicates and to check for completeness and data quality. In case of inconsistencies between the two sources, treatment records were manually checked for clarification and to eliminate true duplicates.

### Study Population

All fresh IVF cycles with successful embryo transfer conducted at the infertility centre of the Bern University Hospital between 2011 and 2016 were considered for this analysis (*n* = 1997). Women older than 42 years at oocyte retrieval (*n* = 62) and cycles stimulated only with clomiphene citrate (*n* = 589) were excluded. Additionally, cycles stimulated by a long agonist protocol were excluded (*n* = 30). Cycles with missing or inconsistent information on stimulation or vitrification that could not be clarified in the records were excluded (*n* = 6).

In total, 1310 cycles were included, 453 NC-IVF and 857 cIVF cycles followed by the transfer of at least one fresh cleavage stage embryo. cIVF cycles with less than four collected oocytes were defined as poor responders (*n* = 333), with four or more oocytes as normal responders (*n* = 524).

As poor ovarian response is associated with higher miscarriage rates [15], cIVF poor responder cycles with less than four collected oocytes were excluded from the main analysis but added for supplementary analysis (*n* = 333). Primary outcomes were defined as the number of amniotic sacs (implantation rate) and the number of live born children per transferred embryo (live birth rate). Amniotic sacs were detected sonographically 4–8 weeks after embryo transfer. The secondary outcome was the number of miscarriages, defined as the number of non-viable amniotic sacs before the 12th week of pregnancy.

The ethics commission of the canton of Bern approved the study on February 26, 2020 (BASEC 2020–00,021).

### Reproductive Treatment

Women with regular menstrual cycles chose their treatment according to their preferences in discussion with the treating physician. In case of anovulation, cIVF is recommended. In cIVF, ≥ 75–300 IU of human menopausal gonadotropins (HMG) per day were used to achieve polyfollicular growth. In most cases, an antagonist protocol and rarely a short agonist protocol was performed. Follicular development was monitored by ultrasound and E2 measurements. When the leading follicle reached a diameter of > 16 mm with a corresponding level of E2, the ovulation was triggered by an injection of human chorionic gonadotropin (HCG). Oocyte retrieval was performed 36 h later with anaesthesia [16, 17].

In NC-IVF, the cycles were monitored by ultrasound measurement of E2 and LH. When one follicle reached a diameter of > 16 mm and E2 levels were expected to be ≥ 700 pmol/L, ovulation was triggered with HCG. Oocyte retrieval was performed 36 h later without anaesthesia [18]. In case of increased risk for premature ovulation, single doses of GnRH antagonists or ibuprofen (400 mg) were administered [19].

The mature oocytes were fertilised by intracytoplasmic sperm injection (ICSI) or in vitro fertilization. The same conditions for embryo culture and lab procedures were applied for all patients, regardless of treatment modality. Zygote but not embryo selection was performed, and all embryos left in culture were transferred at cleavage stage on culture day 2 or 3 using a soft transfer catheter under ultrasound guidance. Supernumerous zygotes were cryopreserved.

Luteal phase support for stimulated cycles was performed with up to 200 mg micronized progesterone twice a day for 9 to 12 weeks after embryo transfer. In NC-IVF cycles, luteal phase support was only recommended if the length of the luteal phase of previous cycles was < 12 days, indicating luteal phase insufficiency [20].

### Statistical Analysis

The baseline characteristics were assessed on cycle level: For the comparison of continuous variables between the two populations, linear regression was used, for the comparison of binary variables, logistic regression was used and for more groups, multinomial logistic regression was used, all with a robust variance estimate clustering on the mother. For the comparison of the main indication of IVF, a chi-square test was applied (Table 1). For implantation, miscarriage and live birth rates, a modified mixed-effect Poisson regression model using a robust variance estimate to achieve rate ratios (RR) and 95% confidence intervals (CI) was conducted [21]. The mixed-effect model was used to account for the dependency of treatment cycles within the same woman [22]. To control for the number of embryos transferred within the same cycle, the number of amniotic sacs and the number of children born was used as count outcomes, while the number of embryos transferred was the exposure to start with in the Poisson regression. For miscarriage rates, the number of amniotic sacs miscarried was used as count outcome and the number of amniotic sacs detected in ultrasound as exposure. The model was adjusted for the following confounding factors: age of mother at oocyte pick-up (in years), parity (yes vs no), main indication for IVF treatment and for type of infertility (primary vs secondary). The main indication for IVF was used as a categorical variable with the following categories: reduced ovarian reserve, tubal factor, endometriosis rASRM stage I/II, endometriosis rASRM stage III/IV (staged according to the revised American Society of Reproductive Medicine (rASRM) endometriosis classification [23]), anovulation, male factor, idiopathic and other reasons. A *p* value of < 0.05 was considered significant. The duration of infertility (in years) was not included in the final model as it did not affect the result. Stratification by parity and by 5-year age group of women was performed for sensitivity analysis in cIVF normal responder and NC-IVF cycles. For supplementary analyses, the baseline characteristics of cIVF poor responders were additionally calculated (Online resource 1). Implantation and live birth rates of embryos transferred in cIVF poor responders were additionally compared to embryos transferred within cIVF normal responders and NC-IVF (Online resource 2) overall and in women aged < 35 years to exclude the effect of age as a reason for poor ovarian response (Online resource 3). All analyses were conducted in STATA Version 16 (StataCorp, College Station, Texas, USA).

**Table 1:** Baseline characteristics of cycles by population (comparison of NC-IVF cycles with cIVF normal responders)

	NC-IVF	cIVF (normal responders)	*p* value
	(*n* = 453)	(*n* = 524)	
Maternal age at oocyte pick-up (years)			0.023^a^
Mean (SD)	35.9 (3.9)	35.1 (4.1)	
Maternal age at oocyte pick-up (group)			0.087^b^
20–24	0 (0.0%)	4 (0.8%)	
25–29	35 (7.7%)	51 (9.7%)	
30–34	119 (26.3%)	158 (30.2%)	
35–39	217 (47.9%)	226 (43.1%)	
40–42	82 (18.1%)	85 (16.2%)	
Duration of infertility (years)			0.009^a^
Mean (SD)	4.1 (2.3)	3.5 (2.3)	
Duration of infertility (group)			0.003^b^
< 1 year	4 (0.9%)	6 (1.1%)	
1–2 years	114 (25.2%)	190 (36.3%)	
3–5 years	240 (53.0%)	248 (47.3%)	
> 5 years	95 (21.0%)	80 (15.3%)	
Primary or secondary infertility			0.688^b^
Primary	343 (75.7%)	389 (74.2%)	
Secondary	110 (24.3%)	135 (25.8%)	
Main indication for IVF treatment			< 0.001^c^
Ovarian reserve	11 (2.4%)	7 (1.3%)	
Tubal factor	49 (10.8%)	52 (9.9%)	
Endometriosis rASRM I/II	42 (9.3%)	37 (7.1%)	
Endometriosis rASRM III/IV	12 (2.6%)	20 (3.8%)	
Anovulation	4 (0.9%)	28 (5.3%)	
Male factor	244 (53.9%)	316 (60.3%)	
Idiopathic	88 (19.4%)	64 (12.2%)	
Other	3 (0.7%)	0 (0.0%)	
Maternal parity at this cycle			0.554^b^
Nulliparous	425 (93.8%)	486 (92.7%)	
Parous	28 (6.2%)	38 (7.3%)	
Oocytes collected at oocyte pick-up			< 0.001^a^
1	435 (96.0%)	0 (0.0%)	
2	18 (4.0%)	0 (0.0%)	
4–6	0 (0.0%)	208 (39.7%)	
7–9	0 (0.0%)	142 (27.1%)	
10–12	0 (0.0%)	100 (19.1%)	
> 12	0 (0.0%)	74 (14.1%)	
Type of fresh transfer			< 0.001^a^
Single-embryo transfer	444 (98.0%)	88 (16.8%)	
Double-embryo transfer	9 (2.0%)	411 (78.4%)	
Triple-embryo transfer	0 (0.0%)	25 (4.8%)	
Number of children born			0.046^a^
No child	393 (86.8%)	442 (84.4%)	
1 child	60 (13.2%)	68 (13.0%)	
2 children	0 (0.0%)	13 (2.5%)	
3 children	0 (0.0%)	1 (0.2%)	

*cIVF* stimulated in vitro fertilization, *NC-IVF* natural-cycle in vitro fertilization, *n* number, *rASRM* revised American Society of Reproductive Medicine endometriosis Classification, *SD* standard deviation

^a^Linear regression with robust standard error clustering on the mother

^b^Logistic regression with robust standard error clustering on the mother

^c^Chi-square test

## Results

**Fig. 1:**
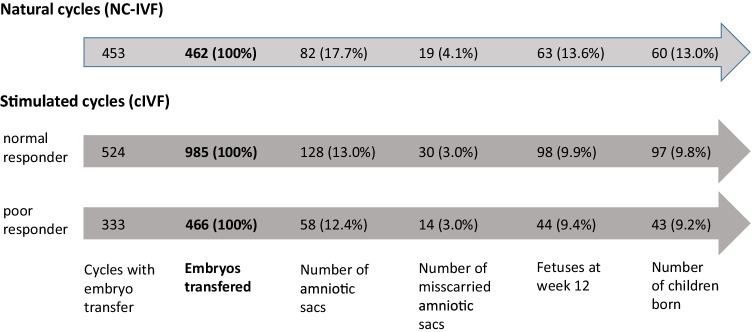
Success rates (crude) per embryo by NC-IVF and cIVF. *Significantly different. **Significantly different in adjusted model. Please note: 100% refers to cycles with successful embryo transfer, which is the study inclusion criteria

In total, 728 couples were included undergoing 1310 IVF treatment cycles, 453 were performed as NC-IVF and 857 as cIVF cycles whereof 524 were in normal responders and 333 cycles were in poor responders (Fig. 1). Of 728 couples, 440 couples contributed one cycle to the analysis and 288 two or more. Most couples, namely 636 (87.4%), had only cycles of the same treatment; 92 couples had cycles of both treatments (12.6%). Of the 524 cycles of cIVF normal responders, 379 treatment cycles were performed as GnRH antagonist and 145 as short GnRH agonist protocols. In total, 1913 embryos were transferred: in NC-IVF 462 (31.9%) embryos were transferred, 444 as single and 18 (9 transfers) as double transfers. In cIVF, 1451 (68.1%) embryos were transferred. In normal responders, a total of 985 embryos were transferred, 88 as single, 822 as double (411 transfers) and 75 (25 transfers) as triple transfers (*p* < 0.001). In poor responders, 466 embryos were transferred, 206 as single, 242 as double (121 transfers) and 18 as triple transfers (6 transfers).

Inclusion criteria and baseline characteristics of the populations were assessed on cycle level. NC-IVF cycles and normal responder cIVF cycles differed regarding age of women, duration of infertility and the couple’s main indication for IVF, whereas type of infertility (primary vs secondary) and women’s parity did not differ (Table 1).

For the following results, NC-IVF was compared to cIVF normal responders (*n* = 977): Implantation and live birth rate per transferred embryo were higher in NC-IVF than in cIVF as presented in Fig. 1 and Table 2. Maternal age and parity were both strongly associated with clinical pregnancy and live birth rates. Both rates were increased in NC-IVF compared to cIVF, mostly in women aged 30–34 years (RR 2.00; 1.28–3.12; *p* = 0.002) in stratified analysis.

**Table 2:** Results per transferred embryo in NC-IVF as compared to cIVF (normal responders)^a^

	Clinical pregnancy^**c**^		Miscarriage^**d**^		Live birth^**c**^	
	RR (95% CI)	*p* value	RR (95% CI)	*p* value	RR (95% CI)	*p* value
Stimulation unadjusted						
NC-IVF	1.37 (1.04–1.81)	0.028	0.99 (0.59–1.65)	0.965	1.33 (0.95–1.86)	0.093
cIVF	1.00 (Ref)		1.00 (Ref)		1.00 (Ref)	
Stimulation adjusted^b^						
NC-IVF	1.42 (1.10–1.84)	0.008	0.90 (0.53–1.54)	0.698	1.38 (1.01–1.88)	0.044
cIVF	1.00 (Ref)		1.00 (Ref)		1.00 (Ref)	
Maternal age at OPU (per year)	0.92 (0.89–0.95)	< 0.001	1.11 (1.04–1.19)	0.002	0.90 (0.87–0.93)	< 0.001
Parity (nulliparous or parous)	4.33 (3.18–5.90)	< 0.001	0.61 (0.29–1.28)	0.193	5.07 (3.55–7.25)	< 0.001
Secondary infertility	0.62 (0.45–0.86)	0.004	1.18 (0.61–2.26)	0.625	0.57 (0.39–0.85)	0.006

*CI* confidence interval, *cIVF* stimulated  IVF, *NC-IVF* natural-cycle IVF, *n* number of cycles, *OPU* oocyte pick-up, *RR* rate ratio

^a^Normal responders defined as > 3 oocytes collected at OPU following gonadotropin stimulation (≥ 75 IU/day)

^b^Model adjusted for maternal age at OPU, parity, secondary or primary infertility and main indication for IVF (RR and 95% CI for main indication for IVF are not presented)

^c^Rate ratios for clinical pregnancy and live birth per embryo transferred

^d^Rate ratios for miscarriage per amniotic sac seen in ultrasound

Miscarriage rates up to 12 gestational weeks were similar in both groups: Of all cycles with a clinical pregnancy (*n* = 186; 80 in NC-IVF and 106 in cIVF), 18 NC-IVF (2.3%) and in 21 cIVF (2.0%) cycles ended in a miscarriage. In NC-IVF, 19 of 82 (23.2%) amniotic sacs and in cIVF 30 of 128 (24.4%) amniotic sacs miscarried. Maternal age was the only associated covariate in the adjusted model (Fig. 1; Table 2).

Sensitivity analysis in parous women (*n* = 56) included 66 cycles, 28 NC-IVF and 38 cIVF of normal responders; the implantation rate of NC-IVF embryos (*n* = 29) was even higher compared to cIVF embryos (*n* = 72): In parous women, 27 NC-IVF embryos were transferred as single and 2 as double transfers. In cIVF 8 embryos were transferred as single, 52 as double transfer and 12 as triple transfers. In NC-IVF, 20 embryos (69.0%) and in cIVF 22 embryos (30.6%) developed into an amniotic sac (RR 2.26; 1.32–3.86; *p* = 0.003). In the adjusted model, the aRR was 2.23 (1.38–3.61; *p* = 0.001) and maternal age (*p* = 0.009) and secondary infertility (*p* = 0.009) were significantly associated with clinical pregnancy rate. In NC-IVF, 2 of 20 (10.0%) amniotic sacs and in cIVF 5 of 22 (22.7%) amniotic sacs miscarried up to 12 gestational weeks. In NC-IVF, one miscarried in the second trimester. The live birth rate per transferred embryo in parous women was higher following NC-IVF; 17 singletons were born out of 29 (58.6%) embryos in NC-IVF compared to 17 (9 singletons and 4 pairs of twins) out of 72 (23.6%) embryos following cIVF (RR 2.48; 95% CI 1.35–4.55; *p* = 0.003; aRR 2.35; 95% CI 1.35–4.10; *p* = 0.003). Results of supplementary analysis including poor responders (*n* = 1310) revealed that poor responders in our sample are older and have a longer time of subfertility. No differences were found between poor and normal responders regarding livebirth rates. For implantation rates, poor responders were slightly attenuated compared to normal responders (online resource 2). Results for miscarriages were difficult to obtain as numbers are low. These results were confirmed in women age < 35 years (online resource 3).

## Discussion

The impact of gonadotropin stimulation on the potential of fresh embryos to implant and to evolve into a clinical pregnancy and live birth was analysed using the models of cIVF and NC-IVF, both without embryo selection. The study revealed that embryos derived from naturally matured follicles as in NC-IVF had a higher implantation potential leading to higher implantation and live birth rates, but miscarriage rates did not differ. These results seemed not to be influenced by the number of oocytes gained at oocyte-pickup and the response to stimulation, as results in poor responders were similar to normal responders within cIVF.

The main strength of the study is the comparison of gonadotropin-stimulated cIVF and unstimulated NC-IVF performed in one centre to reduce the impact of differences in laboratory procedures. Furthermore, IVF treatment is not subsidised in Switzerland and the overall treatment costs per achieved pregnancy are similar in NC-IVF and cIVF [24]; therefore, the risk for selection bias caused by treatment costs is low. The linkage between the registry data and the cohort data improved data quality. Finally, as due to the Swiss law embryo selection was not allowed until 2017, both treatment groups could be compared.

The limitation of the study is its observational design and limited information on treatment details in FIVNAT. Furthermore, some indications such as anovulation differ between NC-IVF and cIVF as NC-IVF requires an ovulatory, regular menstrual cycle. Also, cIVF treatment is heterogeneous using agonist and antagonist protocols and different gonadotropin doses. Since the conduct of a randomized controlled study without embryo selection is not ethically justifiable, the use of observational data is the best possible to analyse the impact of gonadotropins. It is important to be aware that the calculation of success by embryo transferred does not account for early ART failure such as stimulation failure, preterm ovulation and poor response [25].

Women treated by NC-IVF were older, and their duration of infertility was longer; both have been shown to negatively affect pregnancy and live birth rates [12, 26] and still NC-IVF embryos had higher success rates compared to cIVF embryos.

The positive effect of NC-IVF was most pronounced in women aged 30–34 as well as in parous women. Parity is clearly a positive predictive factor in all IVF treatments, but its association with NC-IVF is difficult to disentangle. It seems that NC-IVF might be particularly advantageous in certain subgroups of women.

The better outcome in unstimulated NC-IVF cycles as shown by our study could be due to the following reasons.

First, hormonal stimulation could cause higher aneuploidy rates in embryos, which would lead to higher rates of miscarriages in cIVF. Comprehensive chromosome screening revealed no differences in ploidy status of blastocysts obtained from NC-IVF and cIVF. The euploidy rates were similar in both treatments with an odds ratio (OR) adjusted for the age of the woman of 0.91 (95% CI 0.64–1.18; *P* > 0.05) [27]. These results were also confirmed by the similar miscarriage rate between NC-IVF and cIVF in our study. The aneuploidy rate seems not to be the reason for the differences observed regarding pregnancy and live birth rates.

Second, subtle differences of follicular function due to gonadotropin stimulation might affect the potential of embryos to develop into a clinical pregnancy or a live birth. Follicular fluid of NC-IVF follicles contained different concentrations of immune cells and cytokines. Follicular fluid from NC-IVF contained proportionally less CD45 + leukocytes but more CD8 + cytotoxic T cells than follicular fluid from cIVF [28]. NC-IVF follicular fluid also contained lower levels of vascular endothelial growth factor and marginally increased concentrations of interleukin 8. Furthermore, several follicular hormone concentrations such as LH, testosterone, E2 and AMH are significantly reduced in cIVF follicles, probably due to a lower LH release in cIVF as a result of LH downregulation by GnRH analogues [4]. Follicular fluid AMH concentration correlates with the live birth rate [6] in cIVF and with the clinical pregnancy rate in NC-IVF [7].

Third, a negative hormonal impact on endometrial function could be the reason. Bonagura et al. showed a negative effect of high E2 on placental extra villous trophoblast (EVT) invasion and remodelling of the uterine spiral arteries in baboons [29]. They also revealed a suppression of uterine artery remodelling and expression of extravillous placental vascular endothelial growth factor as well as of α1β1 and α5β1 integrins in baboons [30]. A study comparing biopsies of oocyte donors and naturally cycling women showed an alteration in the expression of genes critical to endometrial remodelling and placentation during the window of implantation [31]. A negatively affected trophoblast differentiation distribution of cell types in the placenta was also shown in mice [32]. In humans, in vitro experiments provided evidence of a negative impact of this supraphysiological effect. Chou et al. found mitochondrial dysfunction in endometrial epithelial cells, and Cottrell et al. described the effects of supraphysiologic levels of E2 on endometrial decidualization and sFlt1 and HOXA10 expression [33, 34]. Higher incidences of disorders of placentation in singletons after IVF in comparison with their non-IVF siblings [35] as well as lower risks in children born after frozen embryo transfer without stimulation in the same cycle support these hypotheses [36]. These effects might be the reason for a lower birth weight and an increased risk to be born small for gestational age [37–39] after fresh cIVF treatments with supraphysiological E2 concentrations.

To what extent each factor contributes to the decrease in the implantation potential of embryos in gonadotropin-stimulated IVF treatments remains an unsolved question. Most likely, interactions of gonadotropin stimulation on different levels within the IVF process can be discussed. Clinically, this study’s findings are in line with other studies regarding the outcome of hypo-, normal and hyper-responders. A meta-analysis showed that mild ovarian stimulation leads to the same outcome than conventional stimulation. Even though fewer oocytes are retrieved and fewer embryos develop after mild stimulation, the proportion of high-grade embryos was similar in all three populations [40]. Furthermore, too high oocyte numbers seem to affect oocyte quality as cumulative live birth rates were reduced, which could explain why our results show no difference between poor and normal responders [2, 3, 41].

In conclusion, this study including only cycles without embryo selection demonstrates that gonadotropin stimulation reduces the potential of fresh embryos to implant. Whether this is due to lower oocyte quality, altered hormonal milieus or lower endometrial receptivity remains unknown. Ideally, this finding would be confirmed in a randomized controlled study.

## Data Availability

Not applicable.
